# Artificial Intelligence and Computational Methods in the Modeling of Complex Systems

**DOI:** 10.3390/e23050586

**Published:** 2021-05-10

**Authors:** Marcin Sosnowski, Jaroslaw Krzywanski, Radomír Ščurek

**Affiliations:** 1 Faculty of Science and Technology, Jan Dlugosz University in Czestochowa, Armii Krajowej 13/15, 42-200 Czestochowa, Poland; j.krzywanski@ujd.edu.pl; 2 Faculty of Safety Engineering, VŠB-Technical University of Ostrava, Lumirova 630/13, 700 30 Ostrava-Vyskovice, Czech Republic; radomir.scurek@vsb.cz

Based on the increased attention, the Special Issue aims to investigate the modeling of complex systems using artificial intelligence and computational methods. More specifically, we invited authors to submit original research articles as well as review articles that provide a novel insight into the analysis and optimization via modeling by artificial intelligence algorithms, etc. The Special Issue of the journal Entropy compiles 19 creative research articles on an innovative application of the above-mentioned modeling methods. The articles in the Special Issue are proof of the importance of the optimization of various complex systems and modeling of their performance. In this Editorial, we provide a brief summary of the papers in this Special Issue, in order of their appearance.

The work by Ślęzak et al. [1] applies the classical Kedem–Katchalsky membrane transport theory to develop a mathematical model dedicated to the simulation of the original concentration volume flux, solute flux characteristics, and S-entropy production in a double-membrane system. Membranes separated the system of two compartments containing homogeneous solutions of one non-electrolytic substance. Based on the model, the original dependencies were calculated for fixed values of transport parameters of membranes.

The study [2] investigates the potential of two new machine learning methods, least-square support vector regression with a gravitational search algorithm and the dynamic evolving neural-fuzzy inference system, for modeling reference evapotranspiration using limited data. The results are compared with the M5 model tree approach. Three indicators are applied to estimate the quality of the models. The obtained results indicate that the temperature or extraterrestrial radiation-based least-square support vector regression with gravitational search algorithm models perform superiorly to the dynamic evolving neural-fuzzy inference system and M5 model tree models in terms of estimating monthly evapotranspiration. Using optimum air temperature and extraterrestrial radiation inputs together generally do not increase the accuracy of the applied methods in the estimation of monthly evapotranspiration.

The analysis of the extreme sea level was carried out within the paper by Khan et al. [3] using tide gauge data of Karachi port station along the Pakistan coast. The research revealed that the magnitudes of the tides usually exceeded the storm surges. The joint probability method and the annual maximum method were used for statistical analysis to find out the return periods of different extreme sea levels. According to the achieved results, the annual maximum and joint probability methods were within the range of 95% confidence. The 10 annual observed sea level maxima showed an increasing trend for extreme sea levels. Tidal analysis, for the Karachi tide gauge data, showed less dependency of the extreme sea levels on the non-tidal residuals. By applying the Merrifield criteria of mean annual maximum water level ratio, it was found that the Karachi coast was tidally dominated and the non-tidal residual contribution was just 10%. 

The paper [4] presents the algorithms for solving the inverse problems on models with the fractional derivative. The analyzed algorithm is based on the real ant colony optimization algorithm and the examples of its application for the inverse heat conduction problem on the model with the fractional derivative of the Caputo type is also presented. The proposed algorithm is compared with the iteration method presented in the literature.

The aim of the work by Lozano et al. [5] is to provide a way of calculating the value of the environmental impacts of an industrial product under different operating conditions. As a case study, the daily production of a newspaper has been chosen. Each process involved in the production was configured with raw material and energy consumption information from production plants, manufacturer data, and existing databases. Four nonlinear regression models have been trained to estimate the impact of a newspaper’s circulation. These nonlinear regression models were trained using the Levenberg–Marquardt nonlinear least-squares algorithm. The mean absolute percentage errors obtained by all the nonlinear regression models tested were less than 5%. Through the proposed correlations, it is possible to obtain a score that reports on the impact of the product for different operating conditions and several types of raw materials.

Zhang et al. [6] studied the least-square support vector regression of the Gaussian–Laplacian mixed homoscedastic and heteroscedastic noise for complicated or unknown noise distributions. The fires one is used to predict the short-term wind speed with historical data. The prediction results indicate that the presented model is superior to the single-noise model, and has a fine performance.

The subject of the study carried out within [7] was the osmotic volume transport of aqueous CuSO_4_ and/or ethanol solutions through a selective cellulose acetate membrane. The effect of concentration of solution components, concentration polarization of solutions, and configuration of the membrane system on the value of the volume osmotic flux in a single-membrane system was examined. Based on the obtained measurement results, the effects of concentration polarization, convection polarization, asymmetry and amplification of the volume osmotic flux, and the thickness of the concentration boundary layers were calculated. Osmotic entropy production was also calculated for solution homogeneity and concentration polarization conditions. Using the thickness of the concentration boundary layers, critical values of the Rayleigh concentration number were estimated between the convective and non-convective state. The operation of this switch indicates the regulatory role of earthly gravity in relation to the membrane transport.

The study [8] by Hodický et al. deals with an experiment on the optimization of NATO ROLE1 medical treatment command according to the key parameters of the numbers of physicians, the number of ambulances, and the distance between ROLE1 and the battlefield. The developed model uses friction data generated from an already executed computer-assisted exercise while employing a constructive simulation to produce offence and defence scenarios on the flow of casualties. The deterministic model of ROLE1, employing a system dynamics simulation paradigm, uses the previously generated casualty flows as the inputs representing human decision-making processes through the recorder computer-assisted exercise events. A factorial experimental design for the ROLE1 model revealed the recommended variants of the ROLE1 structure for both offensive and defensive operations. This study provides novelty in the methodology of casualty estimations involving human decision-making factors as well as the optimization of medical treatment processes through experimentation with the process model.

The paper [9] suggests a new control design based on the concept of synergetic control theory for controlling a one-link robot arm actuated by pneumatic artificial muscles in opposing bicep/tricep positions. The synergetic control design is first established based on known system parameters. However, in real pneumatic artificial muscles-actuated systems, the uncertainties are inherited features in their parameters and hence an adaptive synergetic control algorithm is proposed and synthesized for a pneumatic artificial muscles-actuated robot arm subjected to perturbation in its parameters. The adaptive synergetic laws are developed to estimate the uncertainties and to guarantee the asymptotic stability of the adaptive synergetic controlled pneumatic artificial muscles-actuated system. The work has also improved the performance of proposed synergetic controllers by applying a modern optimization technique based on particle swarm optimization to tune their design parameters towards optimal dynamic performance. The effectiveness of the proposed controllers has been verified via computer simulation. 

This work by Krzywanski et al. [10] presents the results of numerical computations for a large-scale OFz-425 circulating fluidized bed boiler utilizing coal and syngas. Four different operating scenarios are considered, including the reference variant, corresponding to the conventional, mono-combustion of bituminous coal, and three tests involving the replacement of secondary air and part of the coal stream with syngas fed by start-up burners. Pressure, gas velocity, temperature, and carbon dioxide distribution in the combustion chamber are discussed in the paper. The results indicate that the syngas supply leads to an increase in local temperature and carbon dioxide concentrations.

A novel hybrid metaheuristic algorithm is proposed for the dynamic travelling salesman problem in the article by Stodola et al. [11]. The algorithm combines two metaheuristic principles, specifically ant colony optimization and simulated annealing. The significance of the hybridization, as well as the use of knowledge about the dynamic environment, is examined and validated on benchmark instances including small, medium, and large dynamic travelling salesman problems. The results are compared to the four other state-of-the-art metaheuristic approaches with the conclusion that the proposed algorithm significantly outperforms them.

Another contribution to the Special Issue by Krzywanski et al. [12] is focused on the idea of multi-fuel combustion in a large-scale circulating fluidized bed boiler. The article discusses the concept of simultaneous coal and syngas combustion. A comprehensive three-dimensional computational fluid dynamics model is developed, which allows describing complex phenomena that occur in the combustion chamber of the circulating fluidized bed boiler burning coal and syngas produced from coal sludge.

A generalized approximated nonlinear least-squares method by constructing the covariance matrix of residuals is proposed in [13] by Lee et al. The inverse of the covariance matrix is multiplied to the residuals, and it is minimized with respect to the tuning parameters. In addition, an iterative version for the generalized approximated non-linear least-squares namely the max-min G algorithm is considered. In this algorithm, the parameters are re-estimated and updated by the maximum likelihood estimation and the generalized approximated nonlinear least squares, using both computer and experimental data repeatedly until convergence. Moreover, the iteratively re-weighted approximated nonlinear least-squares method was considered for a comparison purpose. Both the bias and variance of estimates obtained from the proposed methods are found to be smaller than those from the approximated nonlinear least squares and the iteratively re-weighted approximated nonlinear least-squares methods. Lastly, an application to a nuclear fusion simulator is illustrated and it is shown that the proposed methods can resolve the abnormal pattern of residuals in the approximated nonlinear least squares.

The article by Studziński [14] submitted to the Special Issue presents several algorithms for controlling water supply system pumps. Ensuring the desired pressure in its recipient nodes, and minimizing energy costs of network operation are the key issues related to the management and operation of water supply networks, apart from the reduction in water losses caused by network failures and ensuring proper water quality. The algorithms presented in the paper have been implemented in an information and communications technology system developed at the Systems Research Institute of the Polish Academy of Sciences and implemented in the waterworks GPW S.A. in Katowice, Poland.

Another paper by Hodický et al. [15], who represents NATO Headquarters Supreme Allied Commander Transformation (Norfolk, VA, USA), presents the developed and executed prototype model with the system dynamics modeling and simulation paradigm. The structure of the model, aggregation mechanism, and shock parametrization methodologies used in the development of the model comprise the scope of the study. The analytic hierarchy process is the methodology that is used for the development of the aggregation mechanism. The parametrized strategic shock input page, analytic hierarchy process-based weighted resilience and risk parameters input pages, one more country insertion to the model, and the decision support system page enhance the capacity of the prototype model. The developed model has the potential to inspire high-level decision-makers dealing with resilience management in other international organizations.

Paper [16] by Gunji et al. investigates the contribution of asynchronous updating to the universal and efficient computation in cellular automata. The computational universality and efficiency in cellular automata are defined and a trade-off relation between the universality and efficiency in cellular automata implemented in synchronous updating is shown. The research shows the significance of asynchronous updating or the timing of computation in robust and efficient computation.

The current state of diffuse irradiance research is discussed by Claywell et al. in [17] and three robust, machine learning models are examined using a large dataset. The machine learning models are a hybrid adaptive network-based fuzzy inference system, a single multi-layer perceptron and a hybrid multi-layer perceptron grey wolf optimizer. The results were evaluated using the mean absolute error, mean error, and the root mean square error. The results showed that the multi-layer perceptron grey wolf optimizer model, followed by the adaptive network-based fuzzy inference system model, provided a higher performance in both the training and the testing procedures.

A novel machine learning method is proposed by Pinter in [18] to tackle real estate modeling complexity. The study explores the potential of call detail records for predicting the real estate price with the aid of artificial intelligence. Several essential mobility entropy factors are used as input variables. The prediction model is developed using the machine learning method of multi-layered perceptron trained with the evolutionary algorithm of particle swarm optimization. Model performance is evaluated using the mean square error, sustainability index, and Willmott’s index. The proposed model showed promising results revealing that the workers’ entropy and the dwellers’ work distances directly influence the real estate price. Furthermore, it is shown that the flow of activities and entropy of mobility are often associated with the regions with lower real estate prices.

An iterative method is proposed in [19] by Seo et al. to address the potential defect of applying the approximated nonlinear least squares method to calibrate the computer model using an emulator such as a Gaussian process model. In the proposed method, the tuning parameters of the simulation model are calculated by the conditional expectation, whereas the maximum likelihood estimation updates the Gaussian process parameters. These expectation-minimization-steps are alternately repeated until convergence using both computer and experimental data. For comparative purposes, another iterative method and a likelihood-based method are considered. Five toy models are tested for a comparative analysis of these methods. According to the toy model study, both the variance and bias of the estimates obtained from the proposed expectation-minimization algorithm are smaller than those from the existing calibration methods. Finally, the application to a nuclear fusion simulator is demonstrated.

We thank all the authors for their excellent contributions and timely submission of their works. We are looking forward to many future developments that will promote all the research activities, making artificial intelligence and computational methods better explainable and more useful.
